# On the Subcutaneous Application of the Ligature for Deep-seated Nævi

**Published:** 1851-04

**Authors:** John Adams

**Affiliations:** Surgeon to the London Hospital, and Lecturer on Anatomy in the School


### SURGERY.

*On the subcutaneous application of the Ligature for deep-seated Nævi.* By John Adams, Esq., F.R.C.S.E., Surgeon to the London Hospital, and Lecturer on Anatomy in the School.—In the treatment of these cases it is of course a primary object to cure the disease without the destruction of the skin which covers it, and which, in many cases, is scarcely involved in the disease. Hence the employment of the seton, the cutting up of the tumor with the couching needle, the injection of irritating lotions, and the introduction of probes armed with nitrate of silver, &c. &c. Nay, even the actual cautery may be used with a view to induce such an amount of inflammation in the cells and blood-vessels of the tumor as shall lead to its complete obliteration.

It has suggested itself to me that a ligature might be so employed as to embrace the whole of the disease without compromising in any manner the vitality of the superjacent skin; and the following is the plan I put into operation in a recent case:—

The disease occurred in a child six months old, and was situated on the right cheek, close to the nose and just beneath the eyelid. The tumor was somewhat oval, its long axis vertical, and, although the disease was principally in the subcutaneous vessels, yet the veins of both upper and lower palpebra were somewhat varicose.

I introduced a long needle armed with a ligature, only slightly curved on the flat towards the point, and carried through the side of the tumor in its long axis, from below upwards; having drawn out the needle on the opposite side, I re-introduced it on the other side of the tumor, at a point on a level with its point of exit, and brought it out opposite the point of its first insertion. If I had now tied the ligature I should have encircled the whole of the tumor, but there would have been great puckering of the skin at the upper and lower edge, whilst the skin on either side would have been free. To obviate this inconvenience, therefore, I cut through the skin above and below, from the point of insertion to the point of exit of the needle, and having tied the ligature firmly, I endeavored to strangulate the tumor.

In this case the success was not complete, and I believe the want of success arose from my having, on the fourth day, divided the knot with a pair of scissors, and removed the ligature before the disease was effectually destroyed.

This method of procedure is founded on the assumption, that subcutaneous nævi are supplied by vessels springing from below rather than by those of the skin. This, however, is not always the case, and I am inclined to think that in the instance just related there were many vessels passing into the tumor from the skin.

I am not aware that this mode of applying the ligature has been put into practice before; it is exceedingly simple, and, as it in nowise compromises the vitality of the skin, I think it deserving of a fair trial.

In another case I certainly should allow the ligature to remain, until, by gentle and gradual traction of its ends, which should be left of about two inches in length, it could be made to cut its way through the base of the tumor, thus completely detaching it from its subjacent connexion.—*London Med. Times.*

